# Sources and pathways of artificial radionuclides to soils at a High Arctic site

**DOI:** 10.1007/s11356-014-3163-6

**Published:** 2014-06-20

**Authors:** E. Łokas, P. Bartmiński, P. Wachniew, J. W. Mietelski, T. Kawiak, J. Środoń

**Affiliations:** 1 Institute of Nuclear Physics, Polish Academy of Sciences, Radzikowskiego 152, 31-342 Krakow, Poland; 2 Maria Curie-Sklodowska University, Lublin, Poland; 3 AGH University of Science and Technology, Krakow, Poland; 4 Institute of Geological Sciences Polish Academy of Sciences, Krakow, Poland

**Keywords:** Artificial radionuclides, Soils, Cryoconites, Proglacial zone, Arctic

## Abstract

Activity concentrations, inventories and activity ratios of ^137^Cs, ^238^Pu, ^239 + 240^Pu and ^241^Am in soil profiles were surveyed in the dry tundra and the adjoining proglacial zones of glaciers at a High Arctic site on Svalbard. Vertical profiles of radionuclide activities were determined in up to 14-cm-thick soil sequences. Additionally, soil properties (pH, organic matter, texture, mineral composition and sorption capacity) were analyzed. Results obtained in this study revealed a large range of activity concentrations and inventories of the fallout radionuclides from the undetectable to the uncommonly high levels (inventories of 30,900 ± 940, 47 ± 6, 886 ± 80 and 296 ± 19 Bq/m^2^ for ^137^Cs, ^238^Pu, ^239 + 240^Pu and ^241^Am, respectively) found in two profiles from the proglacial zone. Concentration of these initially airborne radionuclides in the proglacial zone soils is related to their accumulation in cryoconites that have a large ability to concentrate trace metals. The cryoconites develop on the surface of glaciers, and the material they accumulate is deposited on land surface after the glaciers retreat. The radionuclide inventories in the tundra soils, which effectively retain radionuclides due to high organic matter contents, were comparable to the global fallout deposition for this region of the world. The ^238^Pu/^239 + 240^Pu activity ratios for tundra soils suggested global fallout as the dominant source of Pu. The ^238^Pu/^239 + 240^Pu and ^239 + 240^Pu/^137^Cs activity ratios in the proglacial soils pointed to possible contributions of these radionuclides from other, unidentified sources.

**Electronic supplementary material:**

The online version of this article (doi:10.1007/s11356-014-3163-6) contains supplementary material, which is available to authorized users.

## Introduction

Radioactive isotopes of plutonium, americium and caesium released during the atmospheric testing of nuclear weapons, which peaked in the early 1960s, are detected worldwide, including the High Arctic areas (AMAP 1997). Additionally, the European part of the Arctic is subjected to radioactive contamination from regional sources, such as releases from nuclear industry and nuclear accidents (Dowdall et al. 2003; Gwynn et al. 2004b; Johannessen et al. 2010; Paatero et al. 2012). However, the knowledge of the levels of contamination with the anthropogenic radionuclides in terrestrial compartments of the Arctic environment is limited. Studies concerned with the land areas of the Arctic are less numerous than studies on the radionuclide distribution in the waters, ice and sediments of the Arctic seas (e.g. Baskaran 2005; Masque et al. 2007; Cámara-Mor et al. 2010; Karcher et al. 2010; Zaborska et al. 2010). Due to the scarcity of observations, the sources of radionuclides and their deposition fluxes to soils are not well constrained for the Arctic. For Svalbard, data on anthropogenic radionuclide contents in the terrestrial environment can be found only in the synthetic reports (AMAP 1997, 2004) or in few case studies conducted on tundra soils and peats in the Ny-Ålesund and Hornsund areas (Reszka and Szczypa 1991; Dowdall et al. 2003, 2005a, b, c; Gwynn et al. 2004a, b; Łokas et al. 2013a). Even, less is known about the distribution of radionuclides in the areas deglaciated since the Little Ice Age, which are an important element of the present day landscape of Svalbard (Rachlewicz et al. 2007). These areas are sites of intensive landscape processes associated with reworking of glacigenic sediments and the development of soils (Ziaja 2001, 2004; Lønne and Lyså 2005; Kabała and Zapart 2009, 2012; Irvine-Fynn et al. 2011b). Despite the common use of the anthropogenic radionuclides, mostly ^137^Cs, in studies of geomorphic processes related to redistribution of soils and sediments (Walling 1998; Zapata et al. 2002; Mabit et al. 2008; Łokas et al. 2010; Van Pelt and Ketterer 2013), the applicability of these techniques in the forefields of Arctic glaciers has been explored only for identification of sediment sources and estimation of sedimentation rates in proglacial lakes (Hasholt et al. 2000). The understanding of contents and behaviour of the anthropogenic radionuclides in Arctic soils has also the radioecological importance because soils are a long-term source of radionuclides to the relatively short Arctic food chains. Finally, it can be speculated that the pronounced Arctic climate change affects the fate of radionuclides in soils (Dowdall et al. 2008). Such processes as, for example, changes in atmospheric circulation patterns (Macdonald et al. 2005), prolonged thaw periods and increase in the active layer thickness, changing precipitation regimes and intensification of biological processes may influence transfers of radionuclides to and from the Arctic soils.

**Fig. 1:**
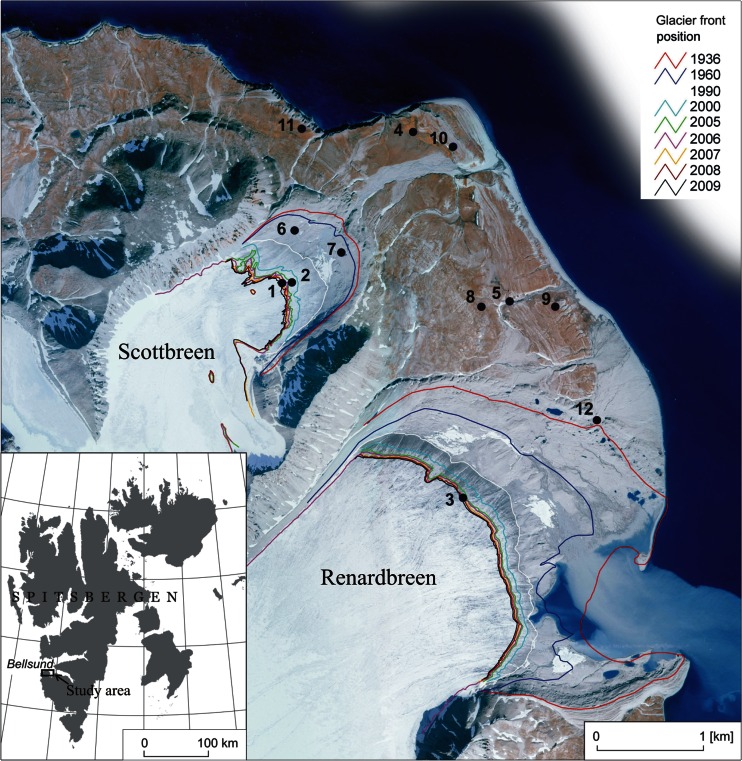
Location of study area, sampling sites and positions of the glacier terminuses from 1936 to 2009. Profiles 2, 3, 6, 7 and 12 were collected from proglacial zones of the glaciers, samples 1 and 5 were alluvial materials, and profiles 4, 5, 8, 9, 10 and 11 were collected from tundra

This paper addresses levels and sources of the fallout radionuclides in soil profiles collected at a site representative for the coastal areas of Western Spitsbergen where strandflats covered by the tundra adjoin the recently deglaciated areas (Fig. 1). An approach applied in this study combines determinations of activity concentrations and ratios of the fallout radionuclides (^238^Pu, ^239 + 240^Pu, ^241^Am and ^137^Cs) in soil profiles with the analyses of soil properties in order to quantify deposition, identify sources, and assess the possibility of post-depositional mobility of these radionuclides. While determinations of radionuclide inventories in soils provide information on their cumulative deposition, only measurements of the vertical patterns of radionuclide activity in soil profiles allow to address temporal variations in deposition fluxes and the post-depositional mobility of radionuclides (Gwynn et al. 2004a, b; Łokas et al. 2013a). Additionally, the analyses of activity ratios allow identification of the ultimate sources of the radionuclide contamination which can be the global fallout from nuclear weapons testing or the regional sources (Salbu 2001; Łokas et al. 2013a; Van Pelt and Ketterer 2013). The soil properties investigated in this study, organic matter content, mineral composition or cation exchange capacity, were shown to influence the soil binding capacity for radionuclides (Cornell 1993; Korobova et al. 2008; Staunton et al. 2002; Solovitch-Vella et al. 2007). This work provides a comprehensive analysis of the occurrence of the anthropogenic radionuclides in the changing Arctic landscape and outlines potential use of these radionuclides as tracers of material transfers in the glacier–proglacial zone systems.

## Materials and methods

### Study area and soil sampling

The investigated area is located in the north-western part of the Wedel Jarlsberg Land (Svalbard) between the snouts of the Scott and Renard glaciers and the seashore. Figure 1 presents the study area, sampling locations and positions of the glacier terminuses from 1936 to 2009 (Zagórski 2005; Zagórski et al. 2008, 2012). The average annual temperature and the average July in the area are about −5 and 5 °C, respectively. Temperatures above 15 °C are noted occasionally. In winter, the average temperatures range from −8 to −16 °C. The amount of precipitation is small, below 400 mm per year. The dominating elements of relief are marine terraces developed as a system of the Pleistocene and Holocene abrasive-accumulation levels. These landforms were and still are shaped by the Renard and Scott glaciers (Zagórski et al. 2008, 2011). The lowest marine terraces consist of sand–gravel and gravel–sand formations. Higher terraces are formed by boulder clay and clay–silt series with sands and gravels. The soil parent material comprises the Quaternary deposits of small thickness including slope covers, glacial deposits and fluvioglacial and marine sediments. The retreat of the glaciers and other climatically induced geomorphic processes have been documented for this area through many studies conducted since the 1980s (Piasecki 1988; Merta et al. 1990; Zagórski et al. 2008; Zagórski 2011; Rodzik et al. 2013). Soils and their origin, distribution, and properties have been described by Klimowicz and Uziak (1988, 1996), Melke and Chodorowski (2006), and Klimowicz et al. (1999, 2009). Fallout radionuclide contents in the Calypsobyen area were studied only by Reszka and Szczypa (1991) who detected contamination from the Chernobyl accident.

During field campaigns conducted in 2007, ten soil profiles (CAL2–CAL4 and CAL6–CAL12) representative for different soil development stages were collected (Fig. 1). The cores of 10–14 cm length were collected by pushing a 9.6-cm internal diameter PCV cylinder into soil. The lengths of the retrieved cores were limited by the thickness of the fine-grained deposits which were underlain by coarse-grained material. The cores were divided into 2-cm-thick subsamples and were dried at 105 °C to constant weight, passed through a 2-mm sieve and prepared for the radionuclide analysis.

The collected profiles represent initial soils (CAL2, CAL3, CAL6, CAL7 and CAL12), tundra soils (CAL4, CAL8, CAL9, CAL10 and CAL11) and fresh alluvial material (CAL1 and CAL5). The initial soils are developing from the recently deposited basal moraines (Kabała and Zapart 2009, 2012) and are characterized by a poor morphological development; the parent material is only slightly modified by the soil-forming processes. The profiles CAL2 and CAL3 were collected from the proglacial zone of the Renard glacier in a closed depression zone and in an old main gate of the glacial river, respectively. The profiles CAL6 and CAL7 were collected from the proglacial zone of the Scott glacier in the vicinity of the lateral and the terminal moraines, respectively, while the profile CAL12 was collected on the terminal moraine of this glacier. The dry tundra profiles CAL4, CAL8, CAL9, CAL10 and CAL11 were classified as brown soils. They were formed as a thin layer underneath sparse vegetation on very permeable sands and gravels of marine terraces (Reszka and Szczypa 1991). Samples of the fresh alluvial material were collected from the ablation moraine of the Scott glacier (CAL1) and from fresh alluvial deposits of a small stream (CAL5). This material was derived from the products of glacial and aeolian weathering washed down and deposited by proglacial streams.

### Radionuclide analyses

Contents of ^137^Cs, ^238^Pu, ^239 + 240^Pu, ^241^Am, as well as soil properties and mineral composition were analyzed in all subsamples. For the ^137^Cs analyses samples were packed into 140-ml plastic containers and their activity measured by high-resolution gamma spectrometry with an HPGe detector. Activities were determined via the ^137m^Ba emission peak at 662 keV. Activities of ^238^Pu, ^239 + 240^Pu and ^241^Am were determined in 10-g aliquots of the samples. Chemical recoveries were determined by alpha spectrometric measurements using ^242^Pu and ^243^Am as the yield tracers. Details of the sequential radiochemical procedure used for the determination of ^239 + 240^Pu, ^238^Pu, and ^241^Am activities are described in detail in the previous publications (Łokas et al. 2010, 2013a). The minimal detectable concentration (MDC) for alpha emitters like Pu isotopes and ^241^Am varied from 0.01 to 0.04 Bq/kg and for ^137^Cs from 0.1 to 1 Bq/kg, depending on the type of detector and on the counting time. Activity concentrations were reported in becquerel per kilogram dry weight. The uncertainties of radionuclide activity concentrations include the measurement uncertainty only and are reported as 1σ counting statistics. The certified soil reference materials (IAEA-375, IAEA-385 and IAEA-447) were used for quality control of measurements. For the IAEA-375, the measured activity of ^239 + 240^Pu (0.27 ± 0.02 Bq/kg) was within the certified range (0.26–0.34 Bq/kg); the activity of ^238^Pu (0.08 ± 0.01 Bq/kg) was at the upper limit of the certified value (0.056–0.085 Bq/kg). The activity of ^137^Cs measured in the IAEA-447 was 431 ± 19 Bq/kg and fell within the uncertainty limits of the certified value (425 ± 10 Bq/kg, decay corrected to 15 November 2009). The activity of ^137^Cs found in IAEA-385 was 38 ± 9 Bq/kg and agreed with the certified value (33 Bq/kg, decay corrected to 1 January 1996). The reference year for all measured Cs-137 activities in soil profiles is 2012. Average activities of blanks measured per each 20 samples were used for background corrections. Radionuclide inventories (Bq/m^2^) in the profiles were calculated by summing the products of radionuclide activity concentrations (Bq/kg) and surface dry masses (kg/m^2^) for each layer. The laboratory performing the analyses has ISO 17025 accreditation for gamma spectrometric measurements and Pu analyses.

### Soil properties

Basic physicochemical properties of soils, contents of organic matter and calcium carbonate, pH, textural composition, were determined for each layer of the soil profiles. Organic matter content was determined as the weight loss on ignition by combustion of 1–2-g aliquots in a muffle furnace for 6 h in 550 °C. Calcium carbonate content was measured volumetrically after treating samples with excess hydrochloric acid. The uncertainties of the CaCO_3_ contents were 5 %. Soil pH in water and in 1 M KCl was measured potentiometrically, with a standard combination glass membrane/silver–silver chloride electrode (Thomas 1996) with the uncertainty of pH 0.002. Analyses of particle size distribution were carried out using a combined sieve–laser method with 1,000 and 500-μm sieves (weight %, 1 phi interval) and FRITSCH Laser Particle Sizer A22C (volume %, ¼ phi interval). Samples with high organic matter content were treated with hydrogen peroxide before the analysis.

Tree profiles, representative for the tundra (CAL4) and the initial (CAL6 and CAL7) soils, were analyzed for mineral composition. Samples were wet ground in methanol for 5 min in McCrone mill with 10 % of zincite as an internal standard (Środoń et al. 2001). Quantitative XRD (X-ray diffraction) mineral compositions were obtained for all 18 samples with the Rietveld-based AutoQuan computer program. Relative uncertainties of the XRD quantitative analysis were evaluated from measurements performed on artificial mineral mixtures and varied from 1 % for the dominant (70–90 %) to 3 % for the minor (10 %) components and were even higher for the trace components. The performance of the quantitative XRD for the carbonates and clay minerals was confirmed by results of chemical analyses (Środoń 2009). The H_2_O and ethylene glycol monoethyl ether (EGME) sorption and cation exchange capacity (CEC) measurements followed the approach of Środoń et al. (2009) and Środoń (2009). The samples were stabilized in a dessicator to a constant weight over a saturated solution of LiNO_3_. Subsequently, the weight of H_2_O released during heating to 200 °C followed by isothermal heating at 200 °C for 0.5 h was measured using a thermobalance. The hot sample was transferred to a dessicator with EGME (2-ethoxyethanol). The mass of adsorbed EGME was measured after equilibration and referred to the 200 °C mass of the dry sample. The colorimetric cobalt hexamine chloride method for the determination of the CEC was used (Orsini and Remy 1976; Bardon et al. 1983).

## Results

### Radionuclide activity concentrations and ratios

**Fig. 2:**
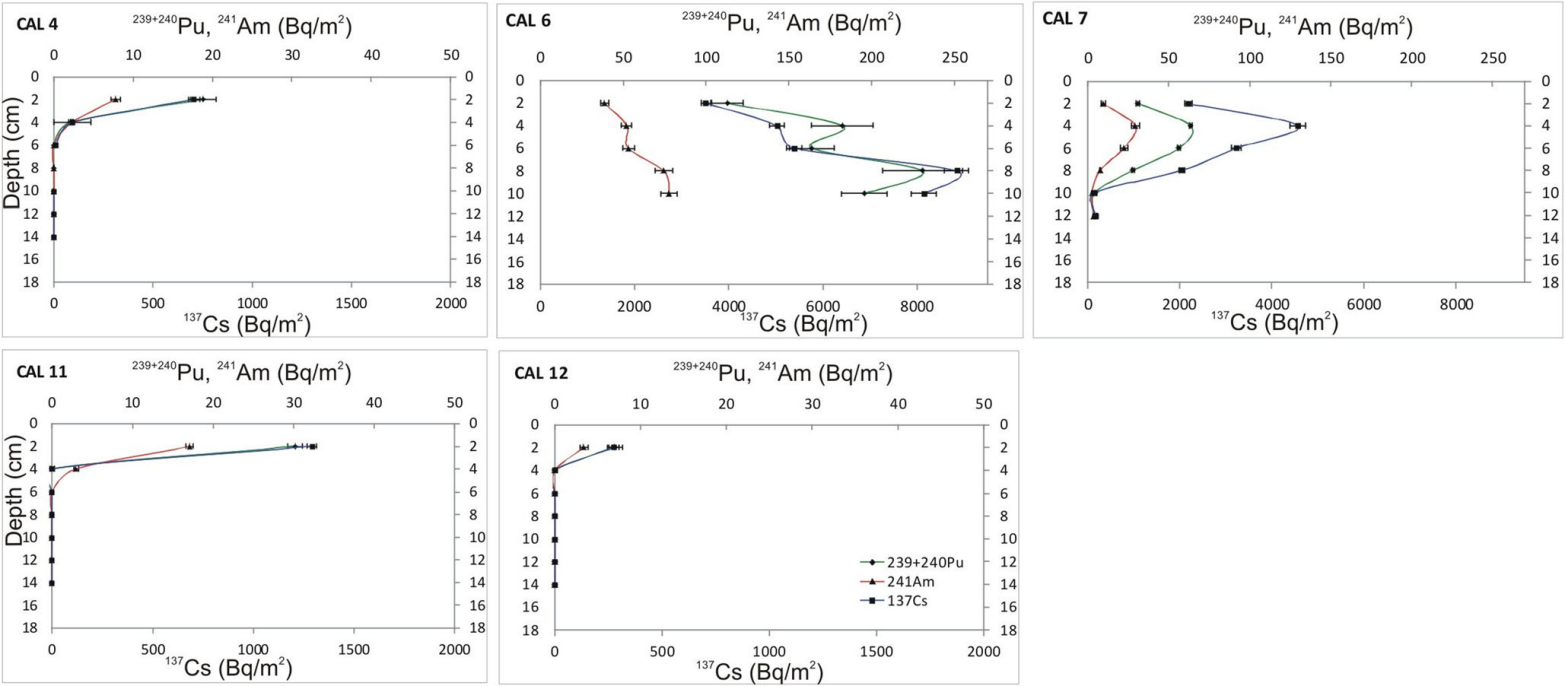
Depth distribution of ^137^Cs, ^239 + 240^Pu and ^241^Am inventories (Bq/m^2^) in five soil profiles

Depth distributions of ^137^Cs, ^239 + 240^Pu and ^241^Am activity concentrations in soil profiles are presented in Table 1 and Fig. 2. The profiles can be divided into three groups with the undetectable, moderate and high levels of radionuclides. Profiles CAL2 and CAL3, as well as the samples of the fresh alluvial material (CAL1 and CAL5) contained undetectable amounts of ^137^Cs (<1 Bq/kg), and activities of other radionuclides in those samples were not determined. In profiles CAL4 and CAL8–CAL12, the radionuclides were contained in the top 4–6 cm of the profiles with maximum activities (up to 180 ± 7 Bq/kg for ^137^Cs, 0.17 ± 0.02 Bq/kg for ^238^Pu, 4.80 ± 0.11 Bq/kg for ^239 + 240^Pu, and 1.99 ± 0.14 Bq/kg for ^241^Am) recorded in the 2-cm-thick topmost layers. Activities found in profiles CAL6 and CAL7 were significantly higher (up to 305 ± 9 Bq/kg for ^137^Cs, 0.40 ± 0.05 Bq/kg for ^238^Pu, 7.96 ± 0.83 Bq/kg for ^239 + 240^Pu and 2.57 ± 0.19 Bq/kg for ^241^Am) and exceeded the detection limits in all subsamples (except for ^238^Pu in two lowest layers of CAL7). The highest activities of all radionuclides in profiles CAL6 and CAL7 were found at the depths of 8 and 6 cm, respectively. Table 1 also presents radionuclide inventories—expressed as the accumulated activity per unit area—for individual soil layers as well as values integrated over the whole profiles. Total inventories differ significantly between the profiles having the lowest values in CAL12 and the extremely high values in CAL6 and CAL7. The inventories calculated for the two latter profiles are by 1–2 orders of magnitude higher than those for the other profiles, reaching in CAL6 the values of 30,900 ± 940 Bq/m^2^ for ^137^Cs, 47 ± 6 Bq/m^2^ for ^238^Pu, 886 ± 80 Bq/m^2^ for ^239 + 240^Pu and 296 ± 19 Bq/m^2^ for ^241^Am.

**Table 1:** Activity concentrations (Bq/kg), inventories (Bq/m^2^) and radionuclide activity ratios for all soil samples

Soil profiles	Depth (cm)	^238^Pu (Bq/kg)	^239 + 240^Pu (Bq/kg)	^241^Am (Bq/kg)	^137^Cs (Bq/kg)	^238^Pu/^239 + 240^Pu	^241^Am/^239 + 240^Pu	^239 + 240^Pu/^137^Cs	^238^Pu (Bq/m^2^)	^239 + 240^Pu (Bq/m^2^)	^241^Am (Bq/m^2^)	^137^Cs (Bq/m^2^)
CAL4-1	2	0.17 ± 0.02	4.80 ± 0.41	1.99 ± 0.14	180 ± 7	0.035 ± 0.005	0.41 ± 0.05	0.027 ± 0.003	0.67 ± 0.01	18.9 ± 1.6	7.8 ± 0.6	708 ± 28
CAL4-2	4	<0.02	0.27 ± 0.03	0.30 ± 0.02	12 ± 1	–	1.11 ± 0.14	0.022 ± 0.003	–	2.1 ± 0.2	2.3 ± 0.2	94 ± 94
CAL4-3	6	<0.02	<0.02	–	1 ± 0.5	–	–	–	–	–	–	11 ± 6
CAL4-4	8	<0.02	<0.02	–	<0.1	–	–	–	–	–	–	–
CAL4-5	10	–	–	–	<0.1	–	–	–	–	–	–	–
CAL4-6	12	–	–	–	<0.1	–	–	–	–	–	–	–
CAL4-7	14	–	–	–	<0.1	–	–	–	–	–	–	–
Inventory (Bq/m^2^)									**0.67** ± **0.1**	**21.0** ± **1.8**	**10.2** ± **0.7**	**813** ± **127**
CAL6-1	2	0.18 ± 0.02	3.18 ± 0.27	1.09 ± 0.07	99 ± 3	0.057 ± 0.008	0.34 ± 0.04	0.032 ± 0.003	6.4 ± 0.7	113 ± 10	38.7 ± 0.7	3,516 ± 107
CAL6-2	4	0.23 ± 0.04	4.72 ± 0.48	1.35 ± 0.08	130 ± 4	0.049 ± 0.010	0.29 ± 0.03	0.036 ± 0.004	8.9 ± 1.5	182 ± 19	52.1 ± 3.1	5,019 ± 154
CAL6-3	6	0.36 ± 0.04	6.11 ± 0.51	1.98 ± 0.13	201 ± 6	0.059 ± 0.010	0.32 ± 0.03	0.030 ± 0.003	9.7 ± 1.1	164 ± 14	53.1 ± 3.5	5,390 ± 161
CAL6-4	8	0.40 ± 0.05	7.96 ± 0.83	2.57 ± 0.19	305 ± 9	0.050 ± 0.008	0.32 ± 0.04	0.026 ± 0.003	11.6 ± 1.4	231 ± 24	74.5 ± 5.5	8,841 ± 261
CAL6-5	10	0.37 ± 0.05	6.82 ± 0.48	2.71 ± 0.17	284 ± 9	0.054 ± 0.008	0.40 ± 0.04	0.024 ± 0.002	10.6 ± 1.4	196 ± 14	77.7 ± 4.9	8,145 ± 258
Inventory (Bq/m^2^)									**47** ± **6**	**886** ± **80**	**296** ± **19**	**30,910** ± **941**
CAL7-1	2	0.06 ± 0.01	1.23 ± 0.10	0.38 ± 0.05	87 ± 3	0.049 ± 0.009	0.31 ± 0.05	0.014 ± 0.001	1.5 ± 0.3	31 ± 3	9.6 ± 1.3	2,189 ± 75
CAL7-2	4	0.08 ± 0.01	1.49 ± 0.12	0.69 ± 0.06	107 ± 4	0.054 ± 0.008	0.46 ± 0.05	0.014 ± 0.001	3.4 ± 0.4	64 ± 5	29.5 ± 2.6	4,567 ± 171
CAL7-3	6	0.12 ± 0.02	2.20 ± 0.15	0.87 ± 0.09	126 ± 4	0.055 ± 0.010	0.40 ± 0.05	0.017 ± 0.001	3.1 ± 0.5	56 ± 4	22.3 ± 2.3	3,232 ± 103
CAL7-4	8	0.06 ± 0.01	0.86 ± 0.06	0.24 ± 0.02	63 ± 2	0.070 ± 0.013	0.28 ± 0.03	0.014 ± 0.001	1.9 ± 0.3	28 ± 2	7.8 ± 0.6	2,039 ± 65
CAL7-5	10	<0.02	0.12 ± 0.01	0.07 ± 0.01	4 ± 1	–	0.58 ± 0.10	0.030 ± 0.008	–	4.5 ± 0.4	2.6 ± 0.4	150 ± 37
CAL7-6	12	<0.02	0.13 ± 0.01	0.10 ± 0.01	5 ± 1	–	0.77 ± 0.10	0.026 ± 0.006	–	4.6 ± 0.4	3.6 ± 0.4	179 ± 36
Inventory (Bq/m^2^)									**9.9** ± **1.5**	**188** ± **14**	**75** ± **8**	**12,355** ± **487**
CAL8-1	2	0.03 ± 0.01	1.43 ± 0.10	0.31 ± 0.02	41 ± 2	0.023 ± 0.004	0.22 ± 0.01	0.035 ± 0.002	0.8 ± 0.3	37.2 ± 0.3	8.1 ± 0.5	1,065 ± 52
CAL8-2	4	<0.02	0.19 ± 0.02	0.04 ± 0.01	5 ± 1	–	0.21 ± 0.06	0.038 ± 0.009	–	3.6 ± 0.4	0.8 ± 0.2	95 ± 19
CAL8-3	6	<0.02	0.05 ± 0.01	<0.06	2 ± 1	–	–	0.025 ± 0.013	–	1.4 ± 0.3	–	57 ± 28
CAL8-4	8	<0.02	<0.02	<0.03	<0.4	–	–	–	–	–	–	–
CAL8-5	10	<0.02	<0.02	<0.21	<1	–	–	–	–	–	–	–
Inventory (Bq/m^2^)									**0.8** ± **0.3**	**42.2** ± **0.9**	**8.8** ± 0.7	**1,217** ± **99**
CAL9-1	2	<0.04	0.40 ± 0.04	0.17 ± 0.02	14 ± 1	–	0.43 ± 0.07	0.029 ± 0.004	–	9.0 ± 0.9	3.8 ± 0.4	315 ± 22
CAL9-2	4	<0.04	0.16 ± 0.01	0.14 ± 0.01	<1	–	0.88 ± 0.08	–	–	4.8 ± 0.3	4.2 ± 0.3	–
CAL9-3	6	<0.04	<0.04	<0.02	<1	–	–	–	–	–	–	–
CAL9-4	8	<0.01	<0.01	<0.02	<1	–	–	–	–	–	–	–
CAL9-5	10	–	–	–	<1	–	–	–	–	–	–	–
CAL9-6	12	–	–	–	<1	–	–	–	–	–	–	–
Inventory (Bq/m^2^)										**13.8** ± **1.2**	**8** ± **0.7**	**315** ± **22**
CAL10-1	2	<0.04	0.68 ± 0.06	0.71 ± 0.05	20 ± 1	–	1.04 ± 0.12	0.034 ± 0.003	–	16.8 ± 1.5	17.6 ± 1.2	495 ± 25
CAL10-2	4	<0.02	0.032 ± 0.005	<0.04	<1	–	–	–	–	0.8 ± 0.3	–	–
CAL10-3	6	<0.02	0.023 ± 0.004	<0.03	<1	–	–	–	–	0.5 ± 0.2	–	–
CAL10-4	8	<0.02	<0.002	<0.02	<1	–	–	–	–	–	–	–
CAL10-5	10	–	–	–	<1	–	–	–	–	–	–	–
Inventory (Bq/m^2^)										**18.2** ± **2.0**	**17.6** ± **1.2**	**495** ± **25**
CAL11-1	2	<0.04	1.24 ± 0.09	0.70 ± 0.04	53 ± 1	–	0.56 ± 0.05	0.023 ± 0.002	–	30.2 ± 2.2	17.1 ± 1.0	1,292 ± 24
CAL11-2	4	<0.03	<0.03	0.110.01	<0.4	–	–	–	–	–	3.0 ± 0.3	–
CAL11-3	6	<0.01	<0.01	<0.03	<1	–	–	–	–	–	–	–
CAL11-4	8	<0.02	<0.02	<0.03	<1	–	–	–	–	–	–	–
CAL11-5	10	–	–	–	<1	–	–	–	–	–	–	–
CAL11-6	12	–	–	–	<0.4	–	–	–	–	–	–	–
CAL11-7	14	–	–	–	<1	–	–	–	–	–	–	–
Inventory (Bq/m^2^)										**31.9** ± **2.2**	**20.1** ± **1.2**	**1,436** ± **24**
CAL12-1	2	<0.02	0.23 ± 0.02	0.11 ± 0.01	9 ± 1	–	0.48 ± 0.06	0.026 ± 0.004	–	7.0 ± 0.6	3.4 ± 0.3	276 ± 31
CAL12-2	4	<0.02	<0.02	< 0.03	<1	–	–	–	–	–	–	–
CAL12-3	6	–	–	–	<0.4	–	–	–	–	–	–	–
CAL12-4	8	–	–	–	<1	–	–	–	–	–	–	–
CAL12-5	10	–	–	–	<1	–	–	–	–	–	–	–
CAL12-6	12	–	–	–	<1	–	–	–	–	–	–	–
CAL12-7	14	–	–	–	<0.3	–	–	–	–	–	–	–
Inventory (Bq/m^2^)										**7.0** ± **0.6**	**3.4** ± **0.3**	**276** ± **31**

Uncertainties are 1σ counting statistics. The inventories of radionuclides were marked in bold in order to make the table more clear

**Fig. 3:**
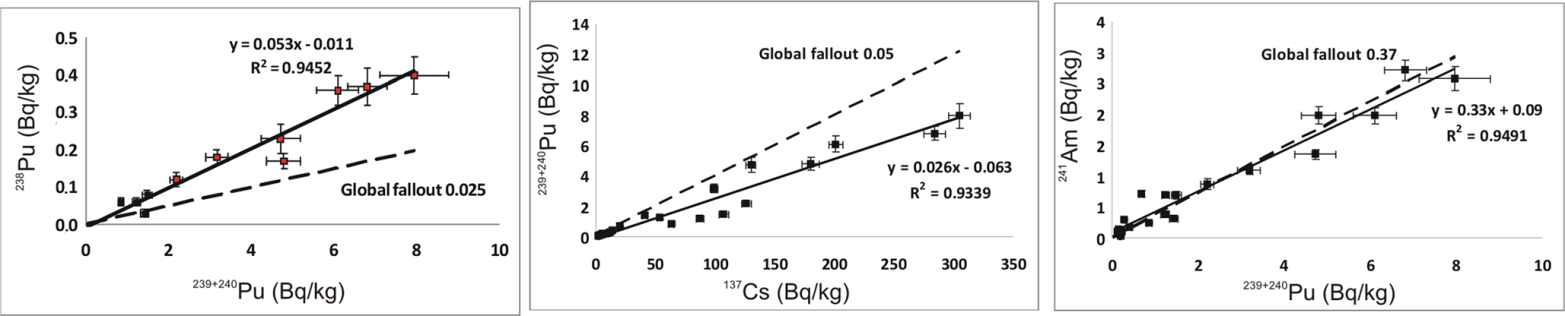
Correlation plots for plutonium isotopes versus caesium and americium for all soil samples

Radionuclide activity ratios are presented in Table 1. The ^238^Pu/^239 + 240^Pu activity ratios were 0.035 ± 0.005 and 0.023 ± 0.004 in the only two samples from profiles CAL4 and CAL8, for which the ratios could be determined and varied between 0.049 ± 0.010 and 0.070 ± 0.013 in profiles CAL6 and CAL7. The arithmetic mean of the all determined ^238^Pu/^239 + 240^Pu ratios was 0.050 ± 0.009. In Fig. 3a, the ^238^Pu and ^239 + 240^Pu activity concentrations are plotted versus each other. Plotting activities of radionuclides in such a manner facilitate interpretation of the obtained activity ratios and their comparison with the reference values (Mietelski et al. 2008; Łokas et al. 2013a). The arithmetic mean of activities may be biased by the outliers which arise from low activity samples whose activities are known with high uncertainties. In this case, the slope of the best fit line (*R* ^2^ = 0.95) shown in Fig. 3a is close to the arithmetic mean of all samples equal to 0.053.

The ^239 + 240^Pu/^137^Cs activity ratios in the soil samples investigated herein ranged between 0.014 ± 0.001 and 0.038 ± 0.009. The arithmetic mean of the ^239 + 240^Pu/^137^Cs activity ratios determined for eight profiles is 0.027 ± 0.009 and is similar to the slope of the best fit line (*R* ^2^ = 0.94) in the ^239 + 240^Pu–^137^Cs correlation plot (Fig. 3b) equal to 0.026. The ^241^Am/^239 + 240^Pu activity ratios in the same profiles ranged between 0.21 ± 0.06 (CAL8) and 1.11 ± 0.14 (CAL4). The average value for all profiles was 0.49 ± 0.06 and was significantly different from the slope of the best fit line (*R* ^2^ = 0.95) in the ^241^Am–^239 + 240^Pu correlation plot (Fig. 3c) equal to 0.33. The difference between both estimates results from very high activity ratios of two samples.

### Physical and chemical properties of soils

The examined soils showed a large variation in organic matter content expressed as loss on ignition (Online Resource 1). Initial soils, in the first stage of development, were characterized by a negligible amount of organic matter (CAL2, CAL3, CAL6, CAL7 and CAL12). The lowest values were measured in the soils lacking clear morphological features of differentiation, which were located in the immediate vicinity of the glacier (CAL2, CAL3). The profiles located farther from the glacier terminus (CAL6, CAL7, CAL12) were subject to the longer periods of organic matter deposition; hence, the organic matter concentrations in those profiles were slightly higher. Organic matter contents in the tundra soils were relatively high, which indicates a certain maturity of these soils. Differences can be observed in the contents of organic matter between the featureless surface-dry tundra (CAL8, CAL11) and morphologically well-developed polygons (CAL9, CAL10) with the better-developed vegetation. Only the surface layers were enriched in humus in the former, while the amount of carbon was high to a depth of several centimetres in the latter.

The pH of soils ranged from slightly acidic to alkaline (Online Resource 1). The highest pH was noted in the least developed soils (CAL2, CAL3, CAL6, CAL7 and CAL12) where concentrations of carbonates were very high, ca. 30–40 % (Online Resource 1). A significantly lower pH, from 5.8 to 7.7 (KCl) generally increasing with depth, was recorded in a relatively mature tundra soils (CAL8–CAL11), which can be associated with a long duration of leaching of the carbonate compounds. The most organic soil (CAL4) was characterized by the lowest pH values (5.5–6.4) decreasing with the depth.

The soils studied belong to the following classes according to the USDA textural soil classification (Soil Survey Staff. Soil Taxonomy 1999): silt loams, silts, sandy loams, loamy sands and sands (Online Resource 1). The finer material, silt loams and silts, was present only in CAL2, the profile closest to the glacier. The profiles CAL6 and CAL7 with the enhanced radionuclide levels have different textures: loamy sand and sand, respectively. The texture of CAL12 varies randomly along the profile from sand to sandy loam. The textural properties of particular soil profiles are not related to the degree of soil development. There is also no relation between the soil textures and radionuclide contents: The profile CAL2 containing the finest fractions has no detectable ^137^Cs, while the predominantly sandy CAL7 is enriched in all radionuclides.

### Mineral composition of soils

All the soils studied contain the same set of detrital minerals: quartz, ordered albite, 2 M_1_ dioctahedral mica, Fe chlorite, potassium feldspar, calcite and dolomite (Online Resource 2). Their proportions in all samples are quite constant, with exception of carbonates, which decrease to trace amounts in the profile CAL4, rich in organic matter, and reveal a good correlation with pH. Relative proportions of minerals within the coarse-grained silicates are the same in the organic and in the mineral soils (Ab/Q in Online Resource 2) indicating lack of chemical weathering of silicates. This conclusion is confirmed by the lack of any measureable differences in the XRD characteristics of chlorite within and between profiles, because chlorite is the least resistant to weathering among the detected silicates. The ratio of fine-grained silicates (Ch + Mica) to coarse-grained silicates (Q + Ab + Ksp) is also constant in individual profiles, indicating their textural homogeneity. A trace of goethite was detected below the upper, most organic-rich sample in the profile CAL4 (Online Resource 2).

**Fig 4:**
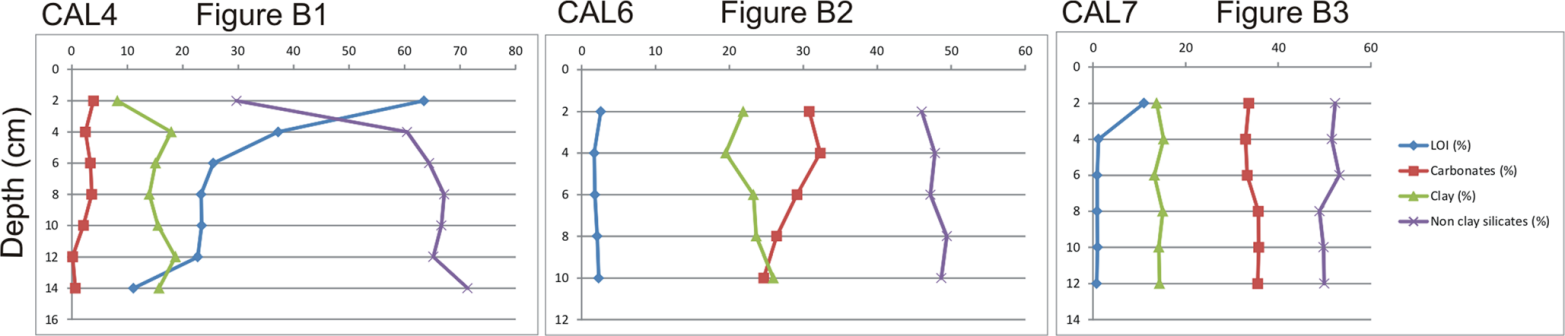
Mineral composition of three soil profiles

Plots of the compositional data for different profiles (Fig. 4) confirm that the main factor differentiating the mineral composition is the dissolution of carbonates induced by organic matter. The profile CAL7 with negligible organic contents has the most undifferentiated mineral composition, including the carbonates. The profile CAL6, where *C*_org_ is significantly higher, displays clear depletion of carbonates in more organic-rich samples, and in the organic soil CAL4, this depletion is very distinct.

Amounts of adsorbed water and EGME are well correlated with the amounts of organic matter while no correlation with clay content is observed. Sorption on the organic matter must be then the controlling mechanism, at least in the organic-rich soil samples. A broad range of EGME values for samples without organics indicates control by the surfaces of clays. Similar relationship exists for CEC, which results in a good correlation of the two values for the organic-poor soils (Online Resource 2). For the organic-rich samples also, CEC is a function of the organic matter content. The values of CEC after removal of organics are similar to the values characterizing the organic-poor soils (Online Resource 2).

## Discussion

### Radionuclide levels

The radionuclide activity concentrations in the tundra profiles CAL4 and CAL8–CAL11 are comparable with the values reported for other Svalbard tundra soils (Dowdall et al. 2005b; Gwynn et al. 2004a, b), while considerably higher activity concentrations of ^137^Cs, ^239 + 240^Pu and ^241^Am (up to 305, 7.96 and 2.71 Bq/kg, respectively) were found in the profiles CAL6 and CAL7 collected from the proglacial zone of the Scott glacier. Similarly high values were observed on Svalbard in the organic layers of soils (Dowdall et al. 2005b) and in peats (Łokas et al. 2013a), but for such organic-rich and low-density deposits, the activity concentrations translate into the whole-profile radionuclide inventories that do not exceed the estimates of atmospheric fluxes for Svalbard (Łokas et al. 2013a). The ^137^Cs inventories in the profiles CAL4 and CAL8–CAL12 (Table 1) are lower than the deposition fluxes (2.2 kBq/m^2^) reported for Svalbard (Hallstadius et al. 1982; Gwynn et al. 2004a, b) and lower than the soil column inventory of 1.6 kBq/m^2^ provided for the Kongsfjord area by Gwynn et al. (2004b). The estimates of ^239 + 240^Pu deposition from atmospheric weapon testing within the 70° N to 80° N latitude belt are 13.3 Bq/m^2^ (Hardy et al. 1973). Holm et al. (1983) reported for Svalbard ^239 + 240^Pu deposition within the range from 14 to 26 Bq/m^2^. The ^239 + 240^Pu inventories exceed these values in the profiles CAL8 and CAL11 where also the ^137^Cs inventories are elevated comparing to the other tundra profiles. The ^238^Pu inventories in the four profiles where that radionuclide was detectable exceed the weapon–testing-derived deposition of 0.3 Bq/m^2^ reported by Hardy et al. (1973) for the 70° N to 80° N latitude belt. There are no available data on the ^241^Am depositional fluxes on Svalbard. Łokas et al. (2013a) found ^241^Am inventories in the peat profiles from SW Svalbard ranging from 4.5 to 27.6 Bq/m^2^. Again, the ^241^Am inventories in CAL4 and CAL8–CAL11 fall within this range, while the inventories in CAL6 and CAL7 exceed it. However, the levels of ^241^Am activity in soils are difficult to interpret because of the in situ ingrowth of this radionuclide from the decay of ^241^Pu (Lee et al. 2005). It must be emphasized that in the profiles CAL6 and CAL7, the inventories of ^137^Cs (30.9 ± 0.9 and 12.4 ± 0.5 kBq/m^2^), ^239 + 240^Pu (886 ± 80 and 188 ± 14 Bq/m^2^) and ^238^Pu (47 ± 6 and 9.9 ± 1.5 Bq/m^2^) are by 1 order of magnitude higher than the above-mentioned reference values. The profile CAL12 collected on the terminal moraine has significant contents of all radionuclides, except the ^238^Pu, but their inventories are lower than in the tundra profiles.

The significant spatial variability of activity concentrations of fallout ^137^Cs and ^239 + 240^Pu in soils was reported for various geographic and climatic settings and attributed to such factors as: links between radionuclide deposition and atmospheric precipitation patterns, spatial heterogeneity and redistribution of snow cover, differences in permeability, density and binding capacities of soils and redistribution of soil particles (Wallbrink et al. 1994; Lettner et al. 2000; Ulsh et al. 2000; Kirchner et al. 2002; Huh and Su 2004). Sampling and analytical uncertainties contribute to this variability as well (Kirchener 2013). It is however unlikely that any of these processes might elevate radionuclide contents to the unusually high levels found in profiles CAL6 and CAL7. Indeed, the typical coefficient of variation (CV) for ^137^Cs inventories in soils, both for the global and Chernobyl fallout, is about 20 % (Sutherland 1996; Pennock 2000; Kirchener 2013). Occasionally, the higher CV values were observed; Ulsh et al. (2000) reported the CV of 40 % for the high altitude sites where ^137^Cs deposition on soils is affected by heterogeneities in snow depth. Huh and Su (2004) reported the CV of 68 % for soils representing very variable textures at the site strongly affected by erosive redistribution of soil. In our case, the CV is 170 % for all profiles and 58 % when CAL6 and 7 are excluded. The elevated radionuclide contents in these two profiles must be thus due to some unaccounted for mechanism capable of concentrating fallout radionuclides.

We hypothesize (Łokas et al. 2013b) that cryoconites—aggregates of mineral and organic substances occurring on glacier surfaces—are the secondary source of the enhanced anthropogenic radioactivity found in the two of our soil profiles. The impurities accumulating on glaciers are mostly of local origin (Bøggild et al. 2010) but contain globally transported anthropogenic contaminants. Indeed, extremely high activity concentrations of fallout radionuclides (up to 140 kBq/kg for ^137^Cs, 188 Bq/kg for ^239 + 240^Pu and 6.27 Bq/kg for ^238^Pu) were reported in cryoconites from Austrian glaciers (Bossew et al. 2007; Tieber et al. 2009). Singh et al. (2013) found high concentrations of heavy metals in cryoconite samples from a glacier located in the Ny-Ålesund area of Svalbard. These lines of evidence suggest that the metallic trace elements, including radionuclides, which are transported in the atmosphere attached to airborne particulate matter, tend to be retained and concentrated in the cryoconite material. The indirect evidence for the role of cryoconites in accumulation of the airborne radionuclides is also available from observations of radionuclide contents in sea-ice sediments (Pfirman et al. 1995; Masque et al. 2003, 2007; Cámara-Mor et al. 2010).

The cryoconites are common in the ablation zones of glaciers, particularly those located at high latitudes and high altitudes (Fountain et al. 2004; MacDonell and Fitzsimons 2008; Hodson et al. 2010). Cryoconite aggregates consist of a mixture of mineral particles, organic substances and living microorganisms, common among them being the filamentous cyanobacteria. The quasi-spherical aggregates increase their size due to the growth of cyanobacteria and physical entrapment of mineral material or by fusion of smaller granules (Takeuchi et al. 2001, 2010). The ability of cryoconite material to retain and concentrate the airborne radionuclides has to be related to metal binding properties of extracellular substances that are excreted by microorganisms in order to immobilize metals and prevent them from entry into the cells. The metals in cationic forms are bound to anionic functional groups such as extracellular polymeric substances (EPS), siderophores and biosurfactants (Gadd 2004; Francis 2007). In fact, the strong affinity of metals, including radionuclides, to those extracellular substances finds application in bioremediation (Bender and Phillips 2004). The cryoconite granules can survive several annual freeze-thaw cycles on glacier surface until they disintegrate after reaching diameter of usually not more than ca. 3 mm, and the resulting fragments have the ability to re-grow into separate granules (Hodson et al. 2010; Takeuchi et al. 2001, 2010). Such recycling of cryoconite material prolongs its exposition to fallout radioactivity and might lead to the build-up of high radionuclide contents, particularly for the material exposed on glacier surfaces to the peak fallout of the 1960s or to fallout from the Chernobyl accident. Isotope ratios of radionuclides found in Alpine cryoconites point to the global fallout as one of the sources of their radioactive contamination (Bossew et al. 2007; Tieber et al. 2009). These observations indicate that cryoconite material may persist on glaciers for periods as long as 40 years.

Because of their low albedo (Takeuchi 2002), cryoconites facilitate melting of ice leading to formation of holes on the glacier surface (Podgorny and Grenfell 1996; MacDonell and Fitzsimons 2008; Cook et al. 2010). The development and fate of cryoconites and cryoconite holes are closely interrelated with meltwater generation and with run-off on glacier surfaces. The cryoconite holes are less frequent in more steep and ablation-prone parts of glaciers where, due to the high volumes and energy of run-off, they cannot develop or have short lifespan (Gribbon 1979; Takeuchi et al. 2000; MacDonell and Fitzsimons 2008, 2012). Once they develop, the cryoconites and cryoconite holes themselves contribute to glacier ablation (Fountain et al. 2004, 2008; MacDonell and Fitzsimons 2008; Bøggild et al. 2010; MacDonell and Fitzsimons 2012). The role of the cryoconite systems in sediment transfers downstream the glacier is, however, not fully understood (MacDonell and Fitzsimons 2008; Irvine-Fynn et al. 2011b). Regardless of the lifespan of individual cryoconite holes, their collapse does not imply removal of cryoconites from glacier surface as the dispersed cryoconite granules initiate formation of new holes Takeuchi et al. (2000). Time-lapse imaging revealed no net movement of granules in the ablation season (Irvine-Fynn et al. 2011a). The retainment of cryoconite material is facilitated by existence of the porous weathering crust (Irvine-Fynn et al. 2012) that develops on the surfaces of non-temperate glaciers (Müller and Keeler 1969). Migration of supraglacial streams was proposed as the most common mechanism of cryoconite evacuation from glacier surfaces (Cook 2012). Once washed down by a stream, the cryoconite granules enter the supraglacial or subglacial drainage system and, even if deposited in the glacier forefront, become diluted by the sediments with low radionuclide contents. The cryoconite holes may be absent near the terminus in high ablation conditions (Gribbon 1979) due to lack of the weathering crust and a continuous flow of melt water. On the other hand, Takeuchi et al. (2000, 2005) reported occurrences of the cryoconite holes near the glacier terminus. Even if the holes are not preserved in the terminal parts of glacier tongues, some fraction of cryoconite granules can be found in deposits of supraglacial streams or dispersed on glacier surfaces (Hodson et al. 2007). Occurrences of the seemingly cryoconite-derived material with high radionuclide contents in the glacier forefront indicate that the cryoconite granules can be retained on glacier surface and deposited at the terminus after ice melts out.

The radionuclide levels differ distinctly between the soil profiles representing different landscape units. The profiles CAL6 and CAL7 with the presumably cryoconite-derived elevated radionuclide levels were collected in the part of the Scott Glacier forefield that was deglaciated between years 1960 and 1990 (Fig. 1). The moderate, typical for Svalbard, levels of radionuclides were found in profiles collected from the dry tundra outside the Holocene extent of the glaciers (CAL4 and CAL8–CAL11) and from the terminal moraine of the Renardbreen (CAL12). The tundra sites, exposed to the atmosphere over the whole period of anthropogenic radionuclide releases, acquired their radionuclide contents primarily from direct atmospheric deposition. Radionuclide inventories in profile CAL12 were lower than in the profiles from tundra pointing to the shorter accumulation period. The terminal moraines of Svalbard valley glaciers were formed during glacier retreat from their maximum extent at the end of Little Ice Age. Sediment deposition at this particular site might began later as the surface of terminal moraines is very unstable due to melting of the ice core and other geomorphic processes. In such conditions, the radionuclides contained in this profile might originate not only from the direct atmospheric deposition but also from the material washed down from the moraine slope. Samples with no detectable radionuclides (CAL1–CAL3 and CAL5) correspond to the fresh alluvial material and to soils collected from the vicinity of the present terminuses of both glaciers. The alluvial material is derived mainly from subglacial weathering and may contain only minute amounts of the debris released from glacial surface.

In general, post-depositional mobility of radionuclides in the High Arctic soils is limited due to a long frost period, low precipitation and lack of bioturbation. Additionally, the CEC and EGME analyses revealed high sorption capacity of the organic-rich tundra soils. The fallout radionuclides were effectively retained in the topmost layers of these profiles. The radionuclides might be mobilized from the proglacial zone profiles, which showed low sorption capacity. Indeed, the activity concentrations in the profile CAL6 remain high down to the bottommost soil layer (Fig. 2) suggesting downwards mobility of radionuclides. However, high activity concentrations at the bottom edge of the profile might also indicate that the accumulation of soil at that particular site began shortly before the peak fallout of the 1960s.

### Activity ratios and sources of radionuclides

Global fallout of radionuclides from the atmospheric nuclear weapons testing that peaked in 1963 was characterized by the ^238^Pu/^239 + 240^Pu activity ratios (for year 1973) of 0.025 (Hardy et al. 1973; Holm et al. 1983). Global contamination with 1 kg of ^238^Pu from a SNAP-9A radioisotopic power source carried by the satellite that disintegrated in the atmosphere in April 1964 (UNSCEAR 2000) increased this ratio (for year 1973) to about 0.03–0.05 (Hardy et al. 1973; UNSCEAR 1982). Decay correction for year 2012 reduces this ratio back to 0.025. The ^238^Pu/^239 + 240^Pu activity ratios found in CAL4 and CAL8 agree with the above ratios while the ratios in CAL6 and CAL7 are consistently higher (Fig. 3, Table 1), suggesting contributions from other than the global fallout sources of plutonium in these two profiles. The possible sources of material with enhanced ^238^Pu/^239 + 240^Pu activity ratios in the European part of the Arctic are as follows: (a) radioactive waste discharged into the Atlantic Ocean from the Sellafield reprocessing plant with the mean value ^238^Pu/^239 + 240^Pu activity ratio of 0.18 (0.03 to 0.36) (MacKenzie et al. 1998; Kershaw et al. 2001), (b) discharges from the Russian nuclear installations and radioactive waste storage sites in the Ob and Yenisey basins with the ^238^Pu/^239 + 240^Pu activity ratios of around 0.19 (Oughton et al. 2004) and (c) Chernobyl accident that released radioactive aerosols with the ^238^Pu/^239 + 240^Pu activity ratios of 0.45–0.60 (Bunzl and Kracke 1990). Because the ^238^Pu/^239 + 240^Pu signatures of these sources are distinctly different than the signature of the global fallout, even relatively small additions of these materials might considerably increase the apparent ^238^Pu/^239 + 240^Pu ratio. Contamination by the radioactive waste from nuclear fuel reprocessing plants is found in the marine environment around Svalbard (Zaborska et al. 2010), and contamination delivered to the Kara Sea by the Ob and Irtysh rivers is transported by sea currents westwards (Cámara-Mor et al. 2010; Masque et al. 2007). However, the transfer of marine contamination to the terrestrial environment may occur only through the deposition of sea spray on land and via animals feeding on sea organisms, mostly birds nesting on the ground. Deposition of contaminated sea spray decreases with the distance from sea shore and should be more pronounced in the nearshore profiles than in the profiles CAL6 and CAL7, located 1 km from the shore behind the ridge of the terminal moraine. Enhanced activities of radionuclides were found in soils below bird colonies on Svalbard (Dowdall et al. 2005c); nevertheless, no bird colonies occur in the vicinity of our sampling sites. The Chernobyl accident may be excluded as a significant source of plutonium contamination on Svalbard since although some amounts of this element were released, they were transported mostly attached to relatively large aerosols (“hot particles”) which were not found further north than the Southern Finland (Paatero et al. 2010; Salminen-Paatero et al. 2014). The elevated ^238^Pu/^239 + 240^Pu activity ratios were also reported by Dowdall et al. (2005b) on Svalbard and Lujaniené et al. (2014) in Lithuania.

There are no consistent differences in the ^239 + 240^Pu/^137^Cs and ^241^Am/^239 + 240^Pu ratios between CAL6–CAL7 and other profiles (Table 1, Fig. 3). The ^239 + 240^Pu/^137^Cs activity ratio averaged over all profiles is 0.027 ± 0.009 and is similar to the slope of regression line in the ^239 + 240^Pu–^137^Cs correlation plot (Fig. 3b) which is found to be 0.026. These values are lower than the decay-corrected value of ~0.05 expected for the year 2012 (Beck and Krey 1983). Similar values (0.027–0.035) were observed on Svalbard by Gwynn et al. (2004a). Cooper et al. (1998) observed even lower ratios (0.014 ± 0.007) in the sea ice entrained sediments in the Arctic Ocean. The enrichment of soils in ^137^Cs relative to plutonium might be due to the contributions from sources other than global fallout or significant fractionation between those radionuclides.

The average value of the ^241^Am/^239 + 240^Pu ratio is 0.49 ± 0.06, while the slope of the linear fit in the ^241^Am–^239 + 240^Pu correlation plot (Fig. 3c) is 0.33. Quantification of the global fallout ^241^Am/^239 + 240^Pu ratio is problematic because of in situ ingrowth of ^241^Am from its parent radionuclide ^241^Pu with the half-time of 14.4 years (Lee et al. 2005; Popov et al. 2010). Consequently, the ^241^Am/^239 + 240^Pu ratio increases with time, and its determination for a given date requires knowledge of both the ^241^Am and ^241^Pu activities. Indeed, the reported ratios vary from 0.3 to 0.65 (Holm et al. 1983; Smith et al. 1997; Gwynn et al. 2005; Łokas et al. 2013a). The usefulness of observed ^241^Am/^239 + 240^Pu ratios to infer the sources of radionuclides is therefore limited.

## Conclusions

The survey of artificial radionuclides (^137^Cs, ^238^Pu, ^239 + 240^Pu, ^241^Am) in shallow soil profiles collected from the dry Arctic tundra and the adjoining proglacial zones revealed the large range of activity concentrations and inventories. At one extreme, ^137^Cs was not detectable in the vicinity of glacier terminuses and in the alluvial deposits of proglacial streams. Lack of ^137^Cs indicates that these materials were derived either from subglacial weathering of bedrock or from reworking of glacial deposits which were previously not exposed to atmospheric fallout of anthropogenic radionuclides. At another extreme, two soil profiles collected in the area uncovered by the retreating glacier between 1960 and 1990 show unusually high activity concentrations and inventories of all radionuclides studied. The elevated contents of the fallout radionuclides found at these two locations can be explained by the action of cryoconites, aggregates of airborne dust, organic matter and microorganisms that develop on glacier surfaces and are deposited in proglacial zones of the retreating glaciers. The ability of cryoconites to retain and concentrate the airborne radionuclides might be related to extracellular substances excreted by microorganisms in order to immobilize metals and prevent them from entry into the cells. The high enrichment of cryoconites with the radionuclides is facilitated by the long persistence of cryoconite granules on glacier surfaces. The intermediate radionuclide levels were found in the soil profiles collected from the dry tundra outside the proglacial areas. The inventories of the tundra profiles fall within the range of atmospheric deposition fluxes reported for Svalbard because the tundra sites were exposed to only the direct atmospheric fallout of the radionuclides.

Of the physical and chemical properties of soils, only the organic matter content influences radionuclide behaviour. The tundra profiles were distinctly enriched in organic matter and depleted in carbonates in comparison with the proglacial zone profiles. The organic-rich topmost layers of the tundra profiles effectively retained the fallout radionuclides. The proglacial zone profiles were composed mostly of the unweathered mineral material and revealed low sorption capacity. The post-depositional mobility of radionuclides in the proglacial zone profiles cannot be excluded; however, its extent in the High Arctic conditions is probably limited.

The ^238^Pu/^239 + 240^Pu and ^239 + 240^Pu/^137^Cs activity ratios in the proglacial soils pointed to possible contributions of these radionuclides from other, unidentified sources. The ^238^Pu/^239 + 240^Pu ratios in the two soils with enhanced radioactivity levels significantly exceed the mean global fallout ratio. Contribution of plutonium from regional sources characterized by the high ^238^Pu/^239 + 240^Pu ratios was excluded because contamination originating from the nuclear installations and contaminated sites in Northern Eurasia is confined to the marine environment around Svalbard.

A survey of fallout radionuclide contents in soils and fine-grained deposits of a proglacial zone can provide insights into the dynamics of deglaciation and pathways of material transfers within the glacier–proglacial zone system. Lack of fallout radionuclides indicates areas recently uncovered by glaciers or accumulations of material originating from such areas. Occurrences of deposits with radionuclide inventories elevated above the atmospheric deposition flux mark areas uncovered after 1960s where deposition of the radionuclide-enriched cryoconite granules occurred. The intermediate levels of radionuclides arise as a result of either limited exposure to the atmospheric fallout or deposition of the reworked deposits.

## Electronic supplementary material

Below is the link to the electronic supplementary material.

ESM 1 (PDF 361 kb)

## References

[CR1] AMAP (1997). Arctic pollution issues: a State of the Arctic Environment Report, Arctic Monitoring and Assessment Programme.

[CR2] AMAP (2004) AMAP Assessment 2002: radioactivity in the Arctic. Arctic Monitoring and Assessment Programme (AMAP), Oslo, Norway

[CR3] Bardon C, Bieber MT, Cuiec L, Jacquin C, Courbot A, Deneuville G, Simon JM, Voirin JM, Espy M, Nectoux A, Pellerin A (1983). Recommandations pour la détermination expérimentale de la capacité d’échange de cations des milieux argileux. Revue de l’ Institut Francais du Petrole.

[CR4] Baskaran M (2005). Interaction of sea ice sediments and surface sea water in the Arctic Ocean: evidence from excess ^210^Pb. Geophys Res Lett.

[CR5] Beck HL, Krey PW (1983). Radiation exposure in Utah from Nevada nuclear tests. Science.

[CR6] Bender J, Phillips P (2004). Microbial mats for multiple applications in aquaculture and bioremediation. Bioresour Technol.

[CR7] Boggild CE, Brandt RE, Brown KJ, Warren SG (2010). The ablation zone in northeast Greenland: ice types, albedos and impurities. J Glaciol.

[CR8] Bossew P, Lettner H, Hubmer A, Erlinger C, Gastberger A (2007). Activity ratios Of Cs-137, Sr-90 and Pu239 + 240 in environmental samples. J Environ Radioactiv.

[CR9] Bunzl K, Kracke W (1990). Simultaneous determination of 238Pu, 239 + 240Pu, 241Pu, 241Am, 241Cm, 244Cm, 89Sr, 90Sr in Vegetation Samples, and application to the Chernobyl-fallout contaminated. Grass J Radioanal Nucl Ch.

[CR10] Cámara-Mor P, Masqué P, Garcia-Orellana J, Cochran JK, Mas JL, Chamizo E, Hanfland C (2010). Arctic Ocean sea ice drift origin derived from artificial radionuclides. Sci Total Environ.

[CR11] Cook J (2012) Microbially mediated carbon fluxes on the surface of glaciers and ice sheets. PhD thesis, University of Sheffield, UK

[CR12] Cook J, Hodson A, Telling J, Anesio AM, Irvine-Fynn T, Bellas C (2010). The mass–area relationship within cryoconite holes and its implications for primary production. Ann Glaciol.

[CR13] Cooper LW, Larsen IL, Beasley TM, Dolvin SS, Grembeier JM, Kelly JM, Scott M, Jonson-Pyrtle A (1998). The distribution of radiocesium and plutonium in sea ice-entrained Arctic sediments in relation to potential sources and sinks. J Environ Radiactiv.

[CR14] Cornell RM (1993). Adsorption of cesium on minerals: a review. J Radioanal Nucl Chem.

[CR15] Dowdall M, Gerland S, Lind B (2003). Gamma-emitting natural and anthropogenic radionuclides in the terrestrial environment of Kongsfjord, Svalbard. Sci. Total Environ.

[CR16] Dowdall M, Gwynn JP, Gabrielsen GW, Lind B (2005). Assessment of elevated radionuclide levels in soils associated with an avian colony in a high arctic environment. Soil Sediment Contam.

[CR17] Dowdall M, Gwynn JP, Moran C, Davids C, O’Dea J, Lind B (2005). Organic soil as a radionuclide sink in a High Arctic environment. J Radioanal Nucl Chem.

[CR18] Dowdall M, Gwynn JP, Moran C, Davids C, O’Dea J, Lind B (2005). Uptake of radionuclides by vegetation at a High Arctic location. Environ Pollut.

[CR19] Dowdall M, Standring W, Shawc G, Strand P (2008). Will global warming affect soil-to-plant transfer of radionuclides?. J Environ Radioactiv.

[CR20] Fountain AG, Tranter M, Nylen TH, Lewis J (2004). Mueller DR (2004) Evolution of cryoconite holes and their contribution to meltwater runoff from glaciers in the McMurdo Dry Valleys. Antarctica J Glaciol.

[CR21] Fountain AG, Nylen TH, Tranter M, Bagshaw E (2008). Temporal variations in physical and chemical features of cryoconite holes on Canada Glacier, McMurdo Dry Valleys, Antarctica. J Geophys Res.

[CR22] Francis AJ (2007). Microbial mobilization and immobilization of plutonium. J Alloy Compd.

[CR23] Gadd GM (2004). Microbial influence on metal mobility and application for bioremediation. Geoderma.

[CR24] Gribbon PWF (1979). Cryoconite holes on Sermikavsak, West Greenland. J Glaciol.

[CR25] Gwynn JP, Dowdall M, Davids C, Selnæs ØG, Lind B (2004). The radiological environment of Svalbard. Polar Res.

[CR26] Gwynn JP, Dowdall M, Lind B (2004b) The radiological environment of Svalbard. Stralevern Rapport 2004:2. Osteras: Norwegian Radiation Protection Authority 1–50

[CR27] Gwynn JP, Dowdall M, Lind B (2005) Plutonium-238, ^239,240^Pu and ^241^Am in terrestrial matrices from Svalbard. In: Environmental radioactivity in the Arctic & Antarctic. Proc. 6^th^ Internat. Conf. 161–164

[CR28] Hallstadius L, Holm E, Persson B, Aarkrog A, Nilsson K (1982) ^137^Cs in the Svalbard area. In: Radiological protection—advances in theory and practice, volume 2. Proc. 3rd Internat. Symp., Inverness, Scotland. (6–11 June) The Society for Radiological Protection 500–505

[CR29] Hardy EP, Krey PW, Volchok HL (1973). Global inventory and distribution of fallout plutonium. Nature.

[CR30] Hasholt B, Walling DE, Owens PN (2000). Sedimentation in arctic proglacial lakes: Mittivakkat Glacier, south-east Greenland. Hydrol Process.

[CR31] Hodson A, Anesio AM, Ng F, Watson R, Quirk J, Irvine-Fynn T, Dye A, Clark C, McCloy P, Kohler J, Sattler B (2007). A glacier respires: quantifying the distribution and respiration CO2 flux of cryoconite across an entire Arctic supraglacial Ecosystem. J Geophys Res.

[CR32] Hodson A, Cameron K, Boggild C, Irvine-Fynn T, Langford H, Pearce D, Banwart S (2010). The structure, biological activity and biogeochemistry of cryoconite aggregates upon an Arctic valley glacier: Longyearbreen, Svalbard. J Glaciol.

[CR33] Holm E, Persson BRR, Halstadius L, Aarkrog A, Dahlgaard H (1983). Radio-cesium and transuranium elements in the Greenland and Barenst Seas. Oceanol Acta.

[CR34] Huh C-A, Su C-C (2004). Distribution of fallout radionuclides (^7^Be, ^137^Cs, ^210^Pb and ^239,240^Pu) in soils of Taiwan. J Environ Radioact.

[CR35] Irvine-Fynn TDL, Barrand NE, Porter PR, Hodson AJ, Murray T (2011). Recent High-Arctic glacial sediment redistribution: a process perspective using airborne lidar. Geomorphology.

[CR36] Irvine-Fynn TDL, Bridge JW, Hodson AJ (2011). In situ quantification of supraglacial cryoconite morphodynamics using time-lapse imaging: an example from Svalbard. J Glaciol.

[CR37] Irvine-Fynn TDL, Edwards A, Newton S, Langford H, Rassner SM, Telling J, Anesio AM, Hodson AJ (2012). Microbial cell budgets of an Arctic glacier surface quantified using flow cytometry. Environ Microbiol.

[CR38] Johannessen OM, Volkov VA, Pettersson LH, Maderich VS, Zheleznyak MJ, Gao Y, Bobylev LP, Stepanov AV, Neelov IA, Tishkov VP and Nielsen SP (2010) Radioactivity and pollution in the Nordic Seas and Arctic Region. Observations, modeling and simulation. Springer

[CR39] Kabała C, Zapart J (2009). Recent, relic and buried soils in the forefield of Werenskiold Glacier, SW Spitsbergen. Pol Polar Res.

[CR40] Kabała C, Zapart J (2012). Initial soil development and carbon accumulation on moraines of the rapidly retreating Werenskiold Glacier, SW Spitsbergen, Svalbard archipelago. Geoderma.

[CR41] Karcher M, Harms I, Standring WJF, Dowdall M, Strand P (2010). On the potential for climate change impacts on marine anthropogenic radioactivity in the Arctic regions. Mar Pollut Bull.

[CR42] Kershaw PJ, Leonard KS, McCubbin D, Aldrige JN, Kudo A (2001). Plutonium: the legacy of Sellafield, plutonium in the environment.

[CR43] Kirchener G (2013). Establishing reference inventories of 137Cs for soil erosion studies: methodological aspects. Geoderma.

[CR44] Kirchner TB, Webb JL, Webb SB, Arimoto R, Schoep DA, Stewart BD (2002). Variability in background levels of surface soil radionuclides in the vicinity of the US DOE waste isolation pilot plant. J Environ Radioact.

[CR45] Klimowicz Z (1999) Soils among rock outliers in the Bellsund region (Spitsbergen). Pol Polar Studies. Lublin 125–131

[CR46] Klimowicz Z, Uziak S (1996). Arctic soil properties associated with micro-relief forms in the Bellsund region (Spitsbergen). Catena.

[CR47] Klimowicz Z, Uziak S (1988). Soil-forming processes and soil properties in Calypsostranda, Spitsbergen. Pol Polar Res.

[CR48] Klimowicz Z, Chodorowski J, Debicki R, Bis M, Kokowski A, Sadowski S (2009). Anthropogenical transformation of soil within the grave-mound. International Agrophysics.

[CR49] Korobova E, Linnik V, Chizhikova N (2008). The history of the Chernobyl ^137^Cs contamination of the flood plain soils and its relation to physical and chemical properties of the soil horizons (a case study). J Geochem Explor.

[CR50] Lee SH, Poviniec PP, Wyse E, Pham MK, Hong GH, Chung CHS, Kim SH, Lee HJ (2005). Distribution and inventories of 90Sr, 137Cs, 241Am and Pu isotopes in sediments of the Northwest Pacific Ocean. Mar Geol.

[CR51] Lettner H, Bossew P, Hubmer AK (2000). Spatial variability of fallout Caesium-137 in Austrian alpine regions. J Environ Radioactiv.

[CR52] Lokas E, Wachniew P, Gąsiorek M, Bartmiński P (2013b) Behaviour of anthropogenic radionuclides in the proglacial environment. Goldschmidt2013 Conference Abstracts Mineral Mag 77 (5): 1660

[CR53] Łokas E, Mietelski JW, Kleszcz K, Tomankiwicz E (2010). A sequential procedure for determining Pu-238, Pu239 + 240, Am-241, Sr-90, U and Th activities in soils and peats from Spitsbergen. Nukleonika.

[CR54] Łokas E, Mietelski JW, Ketterer ME, Kleszcz K, Wachniew P, Michalska S, Miecznik M (2013). Sources and vertical distribution of Cs-137, Pu-238, Pu239 + 240 and Am-241 in peat profiles from southwest Spitsbergen. Appl Geochem.

[CR55] Lønne I, Lyså A (2005). Deglaciation dynamics following the Little Ice Age on Svalbard: implications for shaping of landscapes at high latitudes. Geomorphology.

[CR56] Lujaniené G, Remeikaité-Nikiené N, Garnaga G, Jokšas K, Šilobritiené B, Stankevičius A, Šemčuk S, Kulakauskaité I (2014). Transport of 137Cs, 241Am and Pu isotopes in the Curonian Lagoon and the Baltic Sea. J Environ Radioactiv.

[CR57] Mabit L, Benmansour M, Walling DE (2008). Comparative advantages and limitations of the fallout radionuclides 137Cs, 210Pbex and 7Be for assessing soil erosion and sedimentation. J Environ Radioactiv.

[CR58] Macdonald RW, Harner T, Fyfe J (2005). Recent climate change in the Arctic and its impact on contaminant pathways and interpretation of temporal trend data. Sci Total Environ.

[CR59] MacDonell S, Fitzsimons S (2008). The formation and hydrological significance of cryoconite holes. Prog Phys Geogr.

[CR60] MacDonell S, Fitzsimons S (2012). Observations of cryoconite hole system processes on an Antarctic glacier. Rev Chil Hist Nat.

[CR61] MacKenzie AB, Cook GT, McDonald P, Jones SR (1998). The influence of mixing timescales and re-dissolution processes on the distribution of radionuclides in the northeast Irish Sea sediments. J Environ Radioac.

[CR62] Masque P, Cochran JK, Hebbeln D, Hirschberg DJ, Dethleff D, Winkler A (2003). The role of sea ice in the fate of contaminants in the Arctic Ocean: plutonium atom ratios in the Fram Strait. Environ Sci Technol.

[CR63] Masque P, Cochran JK, Hirschberg DJ, Dethleff D, Hebbeln D, Winkler A, Pfirman S (2007). Radionuclides in Arctic sea ice: tracers of sources, fates and ice transit time scales. Deep-Sea Res I.

[CR64] Melke J, Chodorowski J (2006). Formation of Arctic soils in Chamberlindalen, Bellsund. Spitsbergen Pol Polar Res.

[CR65] Merta T, Ozimkowski W, Osuch D (1990) Evaluation of changes at the forefield of the Scott Glacier based on the photogrammetric data. Wyprawy Geograficzne na Spitsbergen, UMCS, Lublin 51–58

[CR66] Mietelski JW, Olech MA, Sobiech-Matura K, Howard BJ, Gaca P, Zwolak M, Blazej S, Tomankiewicz E (2008). Cs-137, K-40, Pu-238, Pu239 + 240 and Sr-90 in biological samples from King George Island (Southern Shetlands) in Antarctica. Polar Biol.

[CR67] Müller F, Keeler CM (1969). Errors in short-term ablation measurements on melting ice surfaces. J Glaciol.

[CR68] Orsini L, Remy J-C (1976). Utilisation du chlorure de cobaltihexammine pour la determination simultanee de la capacite d’echange et des bases echangeables des sols. Science du Sol.

[CR69] Oughton DH, Skipperud L, Fifield LK, Cresswell RG, Salbu B, Day P (2004). Accelerator mass spectrometry measurement of ^240^Pu/^239^Pu isotope ratios in Novaya Zemlya and Kara Sea sediments. Appl Radiat Isot.

[CR70] Paatero J, Hameri K, Jaakkola T, Jantunen M, Koivukoski J, Saxen R (2010). Airborne and deposited radioactivity from the Chernobyl accident—a review of investigations in Finland. Boreal Environ Res.

[CR71] Paatero J, Vira J, Siitari-Kauppi M, Hatakka J, Holmén K, Viisanen Y (2012). Airborne fission products in the high Arctic after the Fukushima nuclear accident. J Environ Radioac.

[CR72] Pennock DJ (2000). Suitability of redistribution as an indicator of soil quality. Acta Geologica Hispanic.

[CR73] Pfirman SL, Eicken H, Bauch D, Weeks WF (1995). The potential transport of pollutants by Arctic Sea ice. Sci Total Environ.

[CR74] Piasecki J (1988) Przebieg ablacji i strefy glacjalne lodowców Scotta i Renarda (Zachodni Spitsbergen) w sezonie ablacyjnym 1987. Wyprawy Geograficzne na Spitsbergen. UMCS, Lublin 77–91

[CR75] Podgorny IA, Grenfell TC (1996). Absorption of solar energy in a cryoconite hole. Geophys Res Lett.

[CR76] Popov L, Mihailova G, Naidenov I (2010). Determination of activity ratios of Pu-238, Pu-239 + 240, Pu-241, Am-241, Cs-134, Cs-137, and Sr-90 in Bulgarian soils. J Radioanal Nucl Chem.

[CR77] Rachlewicz G, Sztuciński W, Ewertowski M (2007). Post-“Little Ice Age” retreat rates of glaciers around Billefjorden in central Spitsbergen, Svalbard. Pol Polar Res.

[CR78] Reszka M, Szczypa J (1991) The radioactive contamination of southwest Spitsbergen territory. Wyprawy geograficzne na Spitsberen UMCS, Lublin 179–187

[CR79] Rodzik J, Gajek G, Reder J, Zagórski P, Zagórski P, Harasimiuk M, Rodzik J (2013). Glacial geomorphology. Geographical environment of NW part of Wedel Jarlsberg Land (Spitsbergen, Svalbard).

[CR80] Salbu B, Kudo A (2001). Actinides associated with particles. Plutonium in the environment, radioactivity in the environment.

[CR81] Salminen-Paatero S, Paatero J, Jaakkola T (2014). Pu-241 and Pu-241/Pu239 + 240 activity ratio in environmental samples from Finland as evaluated by the ingrowth of Am-241. Boreal Environ Res.

[CR82] Singh SM, Sharma J, Gawas-Sakhalkar P, Upadhyay AK, Naik S, Pedneker SM, Ravindra R (2013). Atmospheric deposition studies of heavy metals in Arctic by comparative analysis of lichens and cryoconite. Environ Monit Assess.

[CR83] Smith JT, Appleby PG, Hilton J, Richardson N (1997). Inventories and fluxes of ^210^Pb, ^137^Cs and ^241^Am determined from the soils of three small catchments in Cumbria, UK. J Environ Radioact.

[CR84] Soil Survey Staff. Soil Taxonomy (1999) A basic system of soil classification for making and interpreting soil surveys. Second Edition. United States Department of Agriculture, Natural Resources Conservation Service. Agric. Handbook 436:1–871

[CR85] Solovitch-Vella N, Pourcelot L, Chen VT, Froidevaux P, Gauthier-Lafaye F, Stille P, Aubert D (2007). Comparative migration behaviour of 90Sr, 239 + 240Pu and 241Am in mineral and organic soils of France. Appl Geochem.

[CR86] Środoń J (2009). Quantification of illite and smectite and their layer charges in sandstones and shales from shallow burial depth. Clay Miner.

[CR87] Środoń J, Drits VA, McCarty DK, Hsieh JCC, Eberl DD (2001). Quantitative X-ray diffraction analysis of clay-bearing rocks from random preparations. Clay Clay Miner.

[CR88] Środoń J, Zeelmaekers E, Derkowski A (2009). The charge of component layers of illite-smectite in bentonites and the nature of end-member illite. Clay Clay Miner.

[CR89] Staunton S, Dumat C, Zsolnay A (2002). Possible role of organic matter in radiocaesium adsorption in soils. J Environ Radioact.

[CR90] Sutherland RA (1996). Caesium-137 soil sampling and inventory variability in reference locations: a literature survey. Hydrol Process.

[CR91] Takeuchi N (2002). Surface albedo and characteristics of cryoconite (biogenic surface dust) on an Alaska glacier, Gulkana Glacier in the Alaska Range. Biulletin of Glaciological Research.

[CR92] Takeuchi N, Kohshima S, Yoshimura Y, Seko K, Fujita K (2000). Characteristics of cryoconite holes on a Himalayan glacier, Yala Glacier Central Nepal. Biulletin of Glaciological Research.

[CR93] Takeuchi N, Kohshima S, Seko K (2001). Structure, formation, and darkening process of albedo-reducing material (cryoconite) on a Himalayan glacier: a granular algal mat growing on the glacier. Arct Antarct Alp Res.

[CR94] Takeuchi N, Matsuda Y, Sakai A, Fujita K (2005). A large amount of biogenic surface dust (cryoconite) on glacier in the Qilian Mountains, China. Biulletin of Glaciological Research.

[CR95] Takeuchi N, Nishiyama H, Li Z (2010). Structure and formation process of cryoconite granules on Ürümqi glacier No. 1, Tien Shan, China. Ann Glaciol.

[CR96] Thomas GW (1996) Soil pH and Soil Acidity. In: Sparks DL, Page AL, Helmke PA, Loeppert RH, Soltanpour PN, Tabatabai MA, Johnston CT, Sumner ME (ed) Methods of soil analysis. Part 3. Chemical methods, Soil Sci. Soc. Am. Book Series: 5. Soil Sci. Soc. Am. Madison pp. 475–490.

[CR97] Tieber A, Lettner H, Bossew P, Hubmer A, Sattler B, Hofmann W (2009). Accumulation of anthropogenic radionuclides in cryoconites on Alpine glaciers. J Environ Radioact.

[CR98] Ulsh B, Rademacher S, Whicker FW (2000). Variations of Cs-137 depositions and soil concentrations between alpine and montane soils in northern Colorado. J Environ Radioactiv.

[CR99] UNSCEAR (1982). Ionizing radiation: sources and biological effects. United Nations Scientific Committee on the effects of Atomic.

[CR100] UNSCEAR (2000). Sources and effects of ionizing radiation.

[CR101] Van Pelt RS, Ketterer ME (2013). Use of anthropogenic radioisotopes to estimate rates of soil redistribution by wind II: the potential for future use of 239 + 240Pu. Aeol Res.

[CR102] Wallbrink PJ, Olley JM, Murray AS (1994). Measuring soil movement using 137Cs: implications of reference site variability. Variability in stream erosion and sediment transport (Proceedings of the Canberra Symposium December 1994). IAHS Publ.

[CR103] Walling DE (1998) Use of 137Cs and other fallout radionuclides in soil erosion investigations: progress, problems and prospects. In: Use of 137Cs in the study of soil erosion and sedimentation. International Atomic Energy Agency Publication IAEA-TECDOC-1028, pp. 39–64

[CR104] Zaborska A, Mietelski JW, Carroll J, Papucci C, Pempkowiak J (2010). Sources and distributions of ^137^Cs, ^238^Pu, ^239,240^Pu radionuclides in the north-western Barents Sea. J Environ Radioact.

[CR105] Zagórski P, Pękala K, Aas HF (2005). NW part of Wedel Jarlsberg Land (Spitsbergen, Svalbard, Norway). Orthophotomap 1:25000.

[CR106] Zagórski P (2011). The shoreline dynamic of Calypsostranda (NW Wedel Jarlsberg Land, Svalbard) during the last century. Pol Polar Res.

[CR107] Zagórski P, Siwek K, Gluza A, Bartoszewski S (2008). Changes in the extent and geometry of the Scott Glacier, Spitsbergen. Pol Polar Res.

[CR108] Zagórski P, Gajek G, Demczuk P (2012). The influence of glacier systems of polar catchments on functioning of the coastal zone (Recherchefjorden, Svalbard). Z Geomorphol.

[CR109] Zapata F, Agudo G, Ritchie JC, Appebly PG, Zapata F (2002). Introduction. Handbook for the assessment of soil erosion and sedimentation using environmental radionuclides.

[CR110] Ziaja W (2001). Glacial recession in Sorkappland and central Nordenskioldland, Spitsbergen, Svalbard, during the 20th century. Arctic Antarctic and Alpine Res.

[CR111] Ziaja W (2004). Spitsbergen landscape under 20th century climate change: Sorkapp Land. AMBIO.

