# Supplementary material for: Sources and pathways of artificial radionuclides to soils at a High Arctic site
Source: Environ Sci Pollut Res Int. 2014 Jun 20;21(21):12479–93. doi: 10.1007/s11356-014-3163-6 (PMC4200352; doi:10.1007/s11356-014-3163-6)
Supplement: Supplementary file 1 — (PDF 361 kb) [file 11356_2014_3163_MOESM1_ESM.pdf]

Electronic Supplementary Material

Sources and pathways of artificial radionuclides to soils at a High Arctic site

Environmental Science and Pollution Research

E. Łokas[1,*], P. Bartmiński[2], P. Wachniew[3], J.W. Mietelski[1], T. Kawiak[4], J. Środoń[4]

[1]Institute of Nuclear Physics Polish Academy of Sciences, Krakow, Radzikowskiego 152, Poland

[2]Maria Curie-Sklodowska University, Lublin, Poland

3 AGH University of Science and Technology, Krakow, Poland

[4]Institute of Geological Sciences Polish Academy of Sciences, Krakow, Poland

*Corresponding Author
Edyta Łokas
Tele: +48 12 6628397
Fax: +48 12 6628392
Email: Edyta.Lokas@ifj.edu.pl

Online Resource 1. Textural properties and chemical properties of soil samples.

Online resource 2. Sorption of water and EGME, cation exchange capacity (CEC) and mineral composition of bulk soil samples. Q - quartz, Ab - albite, Ksp - potassium feldspar, Gol - dolomite, Cal - calcite, Ch - chlorite, Mica - dioctahedral mica (* organic matter removed).

	Textural properties			texture group	pH $_{H2O}$	pH $_{KCl}$	$CaCO_3$ (%)	LOI (%)
	**sand**	**silt**	**clay**					
**CAL2-1**	18.6	74.0	7.4	*silt loam*	8.43	8.27	43.6	-
**CAL2-2**	8.2	83.1	8.7	*silt*	8.47	8.29	42.8	-
**CAL2-3**	19.3	72.6	8.1	*silt loam*	8.49	8.34	42.6	-
**CAL2-4**	45.0	49.4	5.6	*silt loam*	8.45	8.34	43.0	-
**CAL2-5**	73.5	23.5	3.0	*sandy loam*	8.50	8.38	39.1	-
**CAL2-6**	35.5	59.3	5.2	*silt loam*	8.45	8.41	39.4	-
**CAL3-1**	78.0	20.8	1.2	*loamy sand*	8.45	8.41	37.5	-
**CAL3-1**	84.3	14.7	1.0	*loamy sand*	8.42	8.50	36.2	-
**CAL3-1**	87.7	11.5	0.8	*sand*	8.41	8.43	34.8	-
**CAL3-1**	92.4	7.1	0.6	*sand*	8.45	8.35	33.8	-
**CAL3-1**	93.5	6.0	0.5	*sand*	8.45	8.35	40.3	-
**CAL4-1**	49.1	44.9	6.0	*sandy loam*	6.61	6.42	2.0	63.5
**CAL4-2**	65.7	29.4	4.9	*sandy loam*	6.56	6.38	1.2	37.2
**CAL4-3**	81.8	16.5	1.8	*loamy sand*	6.12	5.90	1.9	25.5
**CAL4-4**	79.3	18.9	1.8	*loamy sand*	6.14	5.94	2.4	23.3
**CAL4-5**	73.1	24.7	2.2	*loamy sand*	5.99	5.80	0.7	23.4
**CAL4-6**	70.9	26.8	2.4	*loamy sand*	5.79	5.55	0.0	22.7
**CAL4-7**	75.4	22.8	1.9	*loamy sand*	6.22	5.88	0.0	11.1
**CAL6-1**	59.5	37.2	3.3	*loamy sand*	7.98	7.78	32.1	2.6
**CAL6-2**	73.3	24.5	2.3	*loamy sand*	7.83	7.94	32.8	1.7
**CAL6-3**	70.0	27.6	2.4	*sandy loam*	7.89	7.79	28.2	1.8
**CAL6-4**	65.7	31.5	2.8	*sandy loam*	7.75	7.62	25.5	2.1
**CAL6-5**	56.6	39.9	3.4	*sandy loam*	7.72	7.62	22.9	2.3
**CAL7-1**	86.7	12.2	1.2	*sand*	8.01	7.91	30.0	11.0
**CAL7-2**	85.8	13.0	1.2	*sand*	8.08	8.17	36.6	1.2
**CAL7-3**	88.2	10.7	1.1	*sand*	8.20	8.26	35.1	0.9
**CAL7-4**	90.9	8.3	0.8	*sand*	8.29	8.27	36.1	0.9
**CAL7-5**	96.7	3.0	0.4	*sand*	8.36	8.25	39.4	1.0
**CAL7-6**	95.1	4.5	0.5	*sand*	8.34	8.19	37.9	0.8
**CAL8-1**	80.6	17.1	2.3	*loamy sand*	6.99	6.91	0.0	6.8
**CAL8-2**	72.5	24.5	3.0	*sandy loam*	7.26	6.81	0.0	4.4
**CAL8-3**	72.4	24.8	2.9	*sandy loam*	7.18	6.90	0.0	4.0
**CAL8-4**	75.1	22.2	2.7	*sandy loam*	7.27	6.74	0.0	4.1
**CAL8-5**	69.4	27.2	3.4	*sandy loam*	7.44	6.96	0.0	1.2

**CAL9-1**	88.3	10.3	1.3	*sand*	7.03	6.95	11.3	5.5
**CAL9-2**	85.5	12.9	1.6	*loamy sand*	7.19	6.97	7.5	5.6
**CAL9-3**	86.1	12.4	1.5	*sand*	7.25	6.86	4.4	5.0
**CAL9-4**	87.2	11.5	1.3	*sand*	7.33	6.81	2.3	4.4
**CAL9-5**	87.3	11.3	1.5	*sand*	7.31	7.02	3.0	5.3
**CAL9-6**	88.5	10.1	1.4	*sand*	6.94	6.80	3.3	6.4
**CAL9-7**	88.1	10.4	1.4	*sand*	6.95	6.78	2.8	
**CAL10-1**	89.1	9.5	1.4	*sand*	6.06	5.83	0.0	9.2
**CAL10-2**	86.8	11.5	1.8	*sand*	6.28	5.90	0.0	7.3
**CAL10-3**	84.2	13.8	2.0	*loamy sand*	6.40	5.98	0.0	7.4
**CAL10-4**	83.7	14.3	2.0	*loamy sand*	6.47	6.08	0.0	6.6
**CAL10-5**	86.0	12.3	1.8	*sand*	6.52	6.11	0.0	5.7
**CAL11-1**	82.9	15.6	1.6	*loamy sand*	7.29	7.02	15.5	10.2
**CAL11-2**	86.0	12.9	1.1	*sand*	7.44	7.13	16.6	5.9
**CAL11-3**	87.2	11.6	1.2	*sand*	7.68	7.25	23.4	4.5
**CAL11-4**	88.9	10.1	1.0	*sand*	7.83	7.55	28.2	3.9
**CAL11-5**	89.1	9.9	1.0	*sand*	7.94	7.69	29.9	4.0
**CAL11-6**	90.2	8.9	0.9	*sand*	8.03	7.68	30.1	6.4
**CAL11-7**	88.9	10.1	1.0	*sand*	8.08	7.78	27.7	5.7
**CAL12-1**	71.4	27.0	1.6	*sandy loam*	7.83	7.91	35.4	2.8
**CAL12-2**	86.4	12.6	1.0	*sand*	8.04	8.05	35.5	2.2
**CAL12-3**	75.2	23.3	1.6	*loamy sand*	8.06	8.04	38.7	2.0
**CAL12-4**	57.8	39.9	2.3	*sandy loam*	8.08	8.18	35.5	2.5
**CAL12-5**	61.8	36.2	2.0	*sandy loam*	8.10	8.14	34.4	2.9
**CAL12-6**	73.4	25.0	1.5	*loamy sand*	8.16	8.14	39.5	2.9
**CAL12-7**	93.5	6.0	0.5	*sand*	8.16	8.21	40.9	2.7

Online Resource 1. Textural properties and chemical properties of soil samples.

	H_2O (mg/g)	EGME (mg/g)	CEC (meqv/100g)	Q (%)	Ab (%)	Ksp (%)	Dol (%)	Cal (%)	Ch (%)	Mica (%)	Ch+Mica /Q+Ab+ Ksp	Ab/Q
**CAL4-1**	140.6	35.3	3.62*	26	3	1	3	1	2	6	0.28	0.11
**CAL4-2**	71.2	25.3	15.71	53	7	1	2	0	3	13	0.26	0.13
**CAL4-3**	62.2	19	5.23*	58	6	1	3	0	3	12	0.23	0.11
**CAL4-4**	53.1	16.4	3.59	60	6	1	3	0	3	11	0.21	0.11
**CAL4-5**	60.8	20.4	4.05*	61	5	1	2	0	2	13	0.23	0.08
**CAL4-6**	46	18.4	3.6*	58	6	1	0	0	3	15	0.29	0.11
**CAL4-7**	31	16.9	15.47	64	6	1	0	1	3	13	0.22	0.10
**CAL6-1**	8.3	10.5	3.22	40	4	2	20	11	4	17	0.48	0.11
**CAL6-2**	9.1	8.5	1.09	43	4	1	19	13	5	14	0.41	0.10
**CAL6-3**	8.1	6.8	0.27	42	4	2	18	11	4	19	0.49	0.10
**CAL6-4**	9.6	11.4	3.11	41	7	2	16	11	6	18	0.48	0.17
**CAL6-5**	11.8	11.6	2.28	42	5	2	16	9	6	20	0.53	0.12
**CAL7-1**	13.8	8.1	0.95	46	4	2	19	15	3	10	0.26	0.10
**CAL7-2**	9.6	8.4	0.14	45	4	2	17	16	5	11	0.30	0.10
**CAL7-3**	13.6	8.5	1.09*	47	5	1	17	17	4	9	0.25	0.10
**CAL7-4**	12.4	6.7	0.8*	44	4	1	20	16	3	12	0.31	0.10
**CAL7-5**	10.8	6.8	0.62*	43	4	2	19	17	4	11	0.29	0.09
**CAL7-6**	13.3	6.7	0.4*	45	4	1	19	17	3	11	0.29	0.09

Online resource 2. Sorption of water and EGME, cation exchange capacity (CEC) and mineral composition of bulk soil samples. Q - quartz, Ab - albite, Ksp - potassium feldspar, Gol - dolomite, Cal - calcite, Ch - chlorite, Mica - dioctahedral mica (* organic matter removed).
